# A Concise Review on the Use of Mesenchymal Stem Cells in Cell Sheet-Based Tissue Engineering with Special Emphasis on Bone Tissue Regeneration

**DOI:** 10.1155/2017/2374161

**Published:** 2017-11-05

**Authors:** A. Cagdas Yorukoglu, A. Esat Kiter, Semih Akkaya, N. Lale Satiroglu-Tufan, A. Cevik Tufan

**Affiliations:** ^1^Department of Orthopaedics and Traumatology, School of Medicine, Pamukkale University, Denizli, Turkey; ^2^Department of Forensic Medicine, Forensic Genetics Laboratory, and Department of Pediatric Genetics, School of Medicine, Ankara University, Ankara, Turkey; ^3^Department of Histology and Embryology, School of Medicine, Ankara Yıldırım Beyazıt University, Ankara, Turkey

## Abstract

The integration of stem cell technology and cell sheet engineering improved the potential use of cell sheet products in regenerative medicine. This review will discuss the use of mesenchymal stem cells (MSCs) in cell sheet-based tissue engineering. Besides their adhesiveness to plastic surfaces and their extensive differentiation potential in vitro, MSCs are easily accessible, expandable in vitro with acceptable genomic stability, and few ethical issues. With all these advantages, they are extremely well suited for cell sheet-based tissue engineering. This review will focus on the use of MSC sheets in osteogenic tissue engineering. Potential application techniques with or without scaffolds and/or grafts will be discussed. Finally, the importance of osteogenic induction of these MSC sheets in orthopaedic applications will be demonstrated.

## 1. Introduction

Tissue engineering was emerged as a scientific field in the late 1980s and early 1990s [1–3]. It is defined as “an interdisciplinary field that applies the principles of engineering and the life sciences toward the development of biological substitutes that restore, maintain, or improve tissue function” [2]. Thus, it involves in vitro construction of tissues for implantation into the body to restore, maintain, or improve the form and/or function of a particular tissue and/or organ [4, 5]. The necessities for tissue engineering are defined as “the appropriate levels and sequencing of regulatory signals, the presence and numbers of responsive progenitor cells, an appropriate extracellular matrix, carrier, or scaffold, and an adequate blood supply” [5].

## 2. Tissue Engineering and Cell Sheet Technology

During the course of research in tissue engineering field, direct transplantation of cell suspensions as a cell therapy technique has been considered [6]. However, as reviewed by Shimizu et al. [6], “it is difficult to control the shape, size, and location of the grafted cells” with this technique. In addition, since many cells are lost soon after transplantation, this technique was insufficient to restore the form and/or function of the defected and/or damaged tissue [6–8]. Thus, one of the main research interests of the tissue engineering field has long been the interaction of cells with a variety of biomaterials such as biodegradable polymer scaffolds.

Scaffolds are considered as structures to seed and grow the cells on them, which also serve as carriers for these cells in the process of in vivo implantation [3]. Emerging fields such as genomics, proteomics, drug and/or gene delivery systems, stem cell technologies, biomaterial sciences, nanotechnology, and so forth contributed to the knowledge of interactions between cells and biomaterials. However, the search for an ideal biodegradable biomaterial for cell adhesion, proliferation, and extracellular matrix production is still continuing. Some of the main problems to overcome in this field include “insufficient biological activity, immunogenicity and elevated inflammatory reactions, fluctuating degradation rate, and uncontrollable cell-biomaterial interactions” [9]. Additional problems include “low efficiency of cell attachment and heterogeneous cellular distribution” [9].

An alternative approach to scaffold-based tissue engineering has been the scaffold-free cell sheet-based tissue engineering [7, 8]. The idea of using cultured cells to generate tissues suitable for transplantation goes back to the late 1970s [10]. In the 1980s, cultured autologous human epidermal cells were grown into epithelial skin grafts and used to restore the defects in the epidermis in cases such as severe burns [11], giant congenital nevi [12], and skin ulcers [13]. Studies on the reconstruction of human epidermis with cultured cell sheets continued later on [14, 15].

The so-called “cell sheet” technique was based on culturing cells in hyperconfluency until they form extensive cell-to-cell interactions and produce their own extracellular matrix by which they gain the form of a cell sheet. Kwon and coworkers highlighted in their work the importance of “fabrication of functional tissue constructs using sandwiched layers of cultured cells” and reported the discovery of a temperature-responsive culture dish enabling the rapid detachment and harvesting of cultured cell sheets [16]. The advantages of these temperature-responsive culture surfaces in comparison to enzymatic harvesting of cells from culture dishes were three folds [17, 18]: (1) cell-to-cell connections and extracellular matrix components of cell sheets were well preserved by this technique, (2) adhesive proteins underneath the cell sheets, which play a critical role as an adhesive agent in transferring cell sheets onto other biomaterials or other cell sheets/surfaces/tissues were also well preserved by this technique, and (3) high cell seeding efficacy was also an important advantage of this technique.

In this context, a fabricated single cell sheet may be used for skin, cornea, periodontal ligament, or bladder reconstruction [18]. Several homotypic cell sheets may be layered on top of each other to reconstruct homogenous 3D tissues such as myocardium [18]. Finally, several heterotypic cell sheets may be colayered to construct laminar structures such as liver or kidney [18]. It has been reported that several types of expandable cells are capable of forming transplantable sheets in culture including keratinocytes, retinal pigment epithelial cells, corneal epithelial cells, oral mucosal epithelial cells, urothelial cells, periodontal ligament cells, aortic endothelial cells, corneal endothelial cells, cardiac myocytes, and kidney epithelial cells [19]. In addition, the successful clinical use of cell sheet technology in regenerative applications for the cornea, heart, blood vessels, esophagus, periodontal membrane, functional tendon, and cartilage has been reported [20–24].

The main limitation of cell sheet-based tissue engineering seems to be the possible necrosis inside the cell sheet due to the lack of vascularization [20]. Current work focuses mainly on the construction of 3D vascularized tissues and organs by cell sheet engineering [20].

## 3. Mesenchymal Stem Cells and Their Use in Cell Sheet-Based Tissue Engineering

The integration of stem cell technology and cell sheet engineering improved the potential use of cell sheet products in regenerative medicine. Stem cells are defined as cells which have the capacity to renew themselves and to differentiate into multilineage cells [25]. Based on their origin, stem cells can be classified into three main groups, that is, embryonic stem cells (ESCs), adult stem cells (also named as tissue-specific stem cells, TSSCs), and induced pluripotent stem cells (iPSCs) [25]. The adult stem cells first defined by Friedenstein and coworkers isolated from mouse bone marrow [26] were later named as mesenchymal stem cells (MSCs), in some cases also referred as mesenchymal stromal cells. These multipotent cells have been isolated from almost all tissues including perivascular area [27]. According to the International Society for Cellular Therapy, minimal criteria to define MSCs include the following [28]: (1) these cells adhere to plastic surfaces, (2) in terms of cell surface markers, these cells express CD73, CD90, and CD105, and they lack expression of CD14, CD34, CD45, and HLA-DR, and (3) these cells have the ability to differentiate in vitro into adipocytes, chondrocytes, and osteoblasts. Today, it has been well demonstrated that MSCs have the differentiation potential beyond these three cell types. Ullah et al. have reviewed that “human MSCs have the capacity to differentiate into all the three lineages, that is, ectoderm, mesoderm, and endoderm, with various potency by employing suitable media and growth supplements which initiate lineage differentiation” [25]. Besides their adhesiveness to plastic surfaces and their extensive differentiation potential in vitro, MSCs are also defined as cells that are “easily accessible, culturally expandable in vitro with exceptional genomic stability, and few ethical issues” [25]. With all these advantages, they are extremely well suited for cell sheet engineering.

The sources of MSCs include (but are not limited to) bone marrow, bone tissue, adipose tissue, amniotic fluid, amniotic membrane, dental tissues, endometrium, limb bud, menstrual blood, peripheral blood, placenta and fetal membranes, salivary gland, skin and foreskin, subamniotic umbilical cord lining membrane, synovial fluid, and Wharton's jelly [25].

In terms of long-term culturing of MSCs, it has been demonstrated that culture time and passage number inversely correlate with their differentiation potential. Bonab et al. have cultured human bone marrow-derived MSCs for a mean long-term culture period of 118 days and passaged these cells up to 10 passages [29]. They reported that the average number of population doubling and the mean telomere length decreased with increasing passage number. In addition, these cells started losing their differentiation potential after 6th passage. They concluded that MSCs utilized in regenerative clinical applications should be harvested at earlier passages with shorter in vitro differentiation protocols. Taken into consideration that cell sheets of tissue-derived MSCs can be constructed in an average in vitro culture time of 2-3 weeks using MSCs at 2nd passage [30, 31], it is almost safe to consider that human MSCs can be cultured to form cell sheets in specific media without any abnormalities. The study by See et al. [32], which investigated the effects of the hyperconfluent culture conditions on the multipotentiality of bone marrow-derived MSCs, supports this idea. They have shown that these cell sheets remained viable, they were rich with type I collagen, and they retained their multipotentiality. In addition, there have been studies to improve the formation and also the stemness of stem cell sheets in the literature. Wei et al. reported that Vitamin C treatment promotes in vitro mesenchymal stem cell sheet formation and tissue regeneration by elevating telomerase activity [33]. Jiang et al. reported that methods for cell sheet harvesting include temperature-, electricity-, magnetism-, and pH change-induced methods and suggested a new strategy to obtain MSC sheets using light-induced cell sheet technology [34]. Using this technology, intact MSC sheets were detached from TiO_2_ nanodot-coated quartz substrate after UV365 illumination. Chuah et al. reported that different combinatorial substratum properties, that is, stiffness, roughness, and wettability, on which the MSCs were cultured, were able to influence MSC behavior such as adhesion, spreading, and proliferation during cell sheet formation [35]. Their study concluded that collagen formation within the cell sheet was enhanced on substrates with lower stiffness, whereas, higher hydrophobicity and roughness further assisted the induced chondrogenesis and osteogenesis, respectively. Zhao et al. demonstrated that low oxygen tension and synthetic nanogratings improve the uniformity and stemness of human MSC sheets [36, 37]. In addition to all these applications, the use of oscillatory fluid shear stresses generated by a simple rocking platform has shown to increase collagen secretion of cultured MSCs and apparent collagen organization in their extracellular matrix [38].

Within the last two decades, cell sheets of MSCs have been used in tissue engineering/tissue regeneration of several types of tissues and/or organs including (but are not limited to) the cornea [39], skin [40, 41], cartilage [42, 43], meniscus [44], bone [31, 45–53], tendons [53], cardiac tissue [54–62], periodontal tissue [63, 64], nasal epithelium [65], tooth [66], and blood vessel [67]. Cell sheets of MSCs have also been used in conditions such as wound healing [41], oral ulcers [68], digestive fistula [69], and spinal cord defects [70, 71]. It has also been demonstrated that MSC sheets can induce angiogenesis in ischemic and/or wound tissues [41, 72]. For our purposes, this review will focus on the tissue engineering/tissue regeneration/tissue repair of osteogenic tissue with MSC sheets.

## 4. Mesenchymal Stem Cells and Their Use in Cell Sheet-Based Osteogenic Tissue Engineering

Fractures with large bone defects and/or nonunion are devastating clinical problems in orthopaedics and traumatology. The overall risk of nonunion per fracture was reported as 1.9%; however, for certain fractures, that is, tibial and clavicular fractures, in specific age groups, that is, in young and middle-aged adults, this risk was reported as 9% [73]. Open fractures and fractures with large segmental bone defects increase the ratio of nonunion seen in orthopaedics [74]. Large segmental bone defects often occur also after osteogenic tumor removal.

Several techniques have been suggested to accelerate bone healing process including electrical stimulation, mechanic stimulation, and the use of ultrasound [74, 75]. Autologous, allogenic, or synthetic bone grafts are known as the biological accelerators of the bone healing process [76]. Autologous bone marrow and/or applications of factors such as bone morphogenetic proteins (BMPs) have been used to enhance bone healing [76]. Autologous bone grafts are generally taken from iliac crest and/or tibia. However, harvesting big amounts of graft tissue from donor site causes morbidity such as chronic pain and infection [77, 78]. As an alternative approach, fresh frozen allogenic bone grafts can also be used; however, they increase the risk of transmission of viral diseases and/or induction of immunological reactions in recipients [79, 80]. MSC sheet-based tissue engineering might be a promising field of research for regenerative medicine in terms of overcoming these disadvantages by cell-based therapies.

## 5. The Use of MSC Sheets in Combination with Scaffolds and/or Bone Grafts

Pioneer studies that used MSC sheets in the regeneration of osteogenic tissue considered using these cell sheets like an engineered periosteum tissue around cryopreserved allogenic grafts lacking viable cells [53]. Ouyang et al. assembled MSC sheets onto the demineralized bone grafts by a wrapping technique [53]. They reported that “the assembled structure was cultured for 3 weeks. The macromorphology, histology, and immunohistochemistry of the grafts were evaluated. It was found that MSCs were able to form coherent cellular sheets within 3 weeks. When assembled with demineralized bone matrix, MSC sheets were similar to in situ periosteum and were able to differentiate into the osteochondral lineage.”

Chen et al. investigated the feasibility of bone tissue engineering using a hybrid of MSC sheets and poly (DL-lactic-co-glycolic acid) (PLGA) meshes [52]. They obtained osteogenic sheets of porcine MSCs, which were wrapped onto PLGA meshes resulting in tube-like constructs. These constructs were cultured for 8 weeks in vitro and then implanted to subcutaneous areas of nude rats. They reported that “dense mineralized tissue was formed in subcutaneous sites and the 8-week plants shared similar micro-CT characteristics with native bone. The neotissue demonstrated histological markers for both bone and cartilage, indicating that the bone formation pathway in constructs was akin to endochondral ossification, with the residues of PLGA having an effect on the neotissue organization and formation”. A similar study by Gao et al., who used bone marrow stromal cell sheets assembled with tubular coral scaffolds for long bone regeneration in a mouse model, supported these findings [51]. In addition, when a similar wrapping technique was applied with MSC sheets to structural allografts for segmental bone regeneration, it was possible to repopulate the bone allografts with MSCs and obtain a viable bone construct [46, 50, 81].

There are ongoing investigations to obtain the best results in bone regeneration with MSC sheets. Some of the biomaterials and/or biological agents reported to be combined with MSC sheets to enhance osteogenesis include coral particles [47], ceramics [48], surface-modified titanium and zirconia [82], simvastatin [83], *β*-tricalcium phosphate [84], coumarin-like derivative osthole [85], CD34+ peripheral blood cells [86], a complex of polyethylenimine-alginate nanocomposites plus BMP2 gene [87], nonviral oligonucleotide antimiR-138 delivery to MSC sheets [88], platelet-rich fibrin [89, 90], vitamin C [91], poly(dimethylsiloxane) surface silanization [92], stromal cell-derived factor-1 [93], microRNA-21-loaded chitosan/hyaluronic acid nanoparticles [94], hydroxyapatite particles [90, 95], and Notch activation by Jagged1 in MSC sheet cultures [96].

## 6. Scaffold or Graft-Free Use of MSC Sheets in Bone Regeneration

There have also been studies that utilized MSC sheets without any scaffolds or grafts in bone fracture repair and/or bone regeneration [9, 30, 31, 48, 49, 97].

One of the first studies that investigated the osteogenic differentiation of cultured MSC sheets to obtain bone tissue was reported by Akahane et al. [30]. They fabricated rat bone marrow-derived MSC sheets induced towards the osteogenic differentiation by culture medium supplemented with dexamethasone, L-ascorbic acid phosphate (vitamin C), and *β*-glycerophosphate. After harvesting these sheets, they individually rolled them to obtain tube-like structures and transplanted them into subcutaneous sites on rat thighs without a scaffold to assess whether the sheet could survive and form bone tissue. They reported that X-ray photographs revealed ectopic calcification in the thighs at 6 weeks after sheet transplantation, and histological investigation of dissected sheets after 6 weeks in vivo revealed bone formation. They concluded that “MSCs can be cultured as sheet structures, and the resulting sheets represent osteogenic implants that can be used for hard tissue reconstruction” [30]. Later on, the same group engineered “osteogenic MSC sheets transplanted via injection through a needle and that bone formation results in the injected areas” [49]. These findings were confirmed by studies of Ma et al., who also performed the mechanical testing of the obtained 3D bone tissue, and reported that “the engineered bone exhibited enhanced compressive strength” [9].

The functional use of these osteogenic MSC sheets described previously [30] in a rat nonunion model was reported by Nakamura et al. [31]. They investigated the effects of cell sheets on the healing process after transplanting them onto fractured femurs without a scaffold, that is, they wrapped the sheets around the fracture site. They reported that “X-ray photographs and histological sections showed callus formation around the fracture site in the cell sheet-transplanted group (sheet group). Bone union was obtained in the sheet group at 8 weeks. By contrast, the control group (without sheet transplantation) showed nonunion of the femur” [31]. Thus, they concluded that “cell sheet transplantation can contribute to hard tissue reconstruction in cases involving nonunion, bone defects, and osteonecrosis” [31].

Another functional study utilizing injectable osteogenic MSC sheet fragments in a rabbit distraction osteogenesis model was reported by Ma et al. [97]. In this study, after mandibular osteotomy, osteogenic MSC sheet fragments were injected into the distraction areas and new bone formation was evaluated in time. They reported that “injection of bone marrow stromal cell sheet fragments promotes bone formation in distraction osteogenesis and indicates a promising approach to shorten the treatment period of osteodistraction” [97].

## 7. Demonstration of the Importance of In Vitro Osteogenic Induction of MSC Sheets in Bone Regeneration Applications

As discussed so far in this review, the search to improve the composition of the MSC sheets used in applications for bone regeneration has not been concluded yet. In this context, more than one parameter has been taken into consideration such as in vitro culture period, the composition of the culture medium, effectiveness of the cell sheet with or without scaffolds/grafts, and also the size of the transplantable sheet in terms of handling the larger structural bone defects.

It has been well accepted that “The standard procedure for the osteogenic differentiation of multipotent stem cells is treatment of a confluent monolayer with a cocktail of dexamethasone, ascorbic acid, and *β*-glycerophosphate” [98]. Thus, it has been reported that these MSC sheets are constructed with early passages of MSCs seeded at 1–5 × 10^4^ cells/cm^2^ onto 10 cm culture dishes and cultured under osteogenic conditions with media consisting of the basic medium (MEM/DMEM with 10–15% fetal bovine serum, 100 U/ml penicillin, 100 *μ*g/ml streptomycin, and 2 mM L-glutamine) supplemented with 50 *μ*M L-ascorbic acid, 10 mM *β*-glycerophosphate, and 100 nM dexamethasone for 2-3 weeks [9, 30, 31]. The individual mechanisms of effects of the components of this triple osteogenic cocktail have also been described [98]. In this context, Langenbach and Handschel [98] have reported that “Dexamethasone induces Runx2 expression by FHL2/*β*-catenin-mediated transcriptional activation and that dexamethasone enhances Runx2 activity by upregulation of TAZ and MKP1. Ascorbic acid leads to the increased secretion of collagen type I (Col1), which in turn leads to increased Col1/*α*2*β*1 integrin-mediated intracellular signaling. The phosphate from *β*-glycerophosphate serves as a source for the phosphate in hydroxylapatite and in addition influences intracellular signaling molecules.”

Based on this knowledge, a preliminary study by our group investigated the effects of these osteogenic supplements in different combinations within the culture medium on the structure of the MSC sheets. The procedures used in this study were approved by the Ethical Review Committee of the Pamukkale University, School of Medicine, Denizli, Turkey. Our group had experience with human [99, 100], chicken [101], and rat [102] trabecular bone-derived MSCs. Thus, stocks from the previously isolated and characterized trabecular bone-derived rat MSCs [102] were used in this study. Cell sheets from these MSCs were constructed under the conditions described earlier [9, 30, 31].

**Figure 1:**
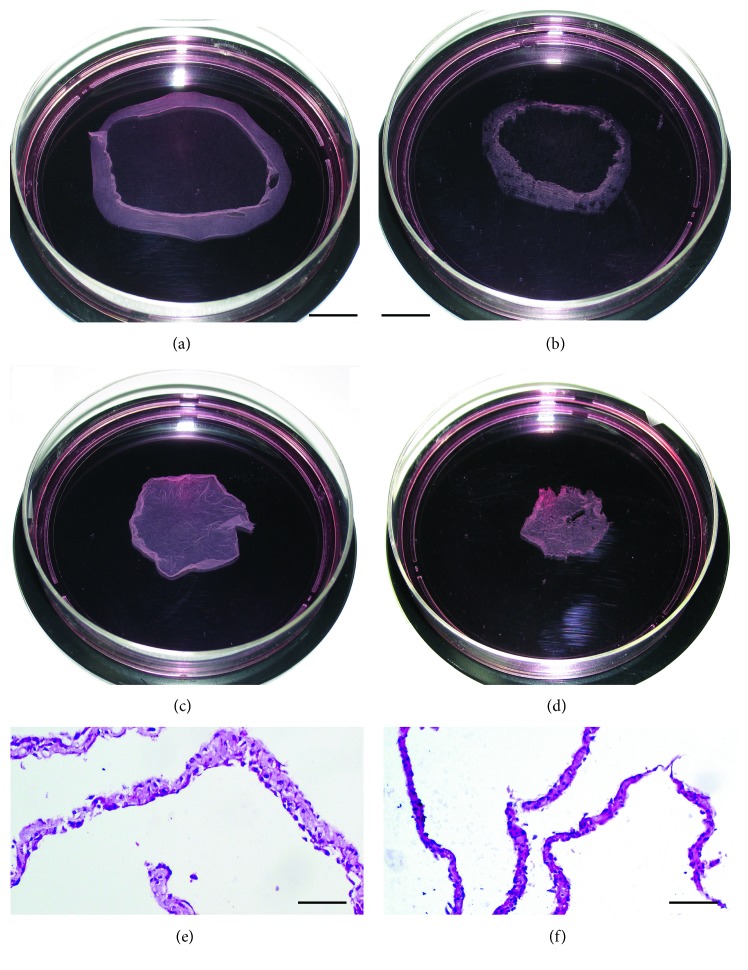
The gross and microscopic appearances of MSC sheets. MSC sheet induced with ascorbic acid before (a) and after (c) detachment from the culture dish. MSC sheet induced with standard osteogenic cocktail before (b) and after (d) detachment from the culture dish. Shrinkage in both groups of cell sheets was observed after complete detachment from the culture dish. The surface area of MSC sheet induced with ascorbic acid was larger than that of MSC sheet in the osteogenic group. In addition, as seen in histological examination, MSC sheet induced with ascorbic acid was thicker (e) than the MSC sheet in the osteogenic group (f). Scale bar = 2 cm for (a, b, c, d), scale bar = 100 *μ*m for (e, f).

**Figure 2:**
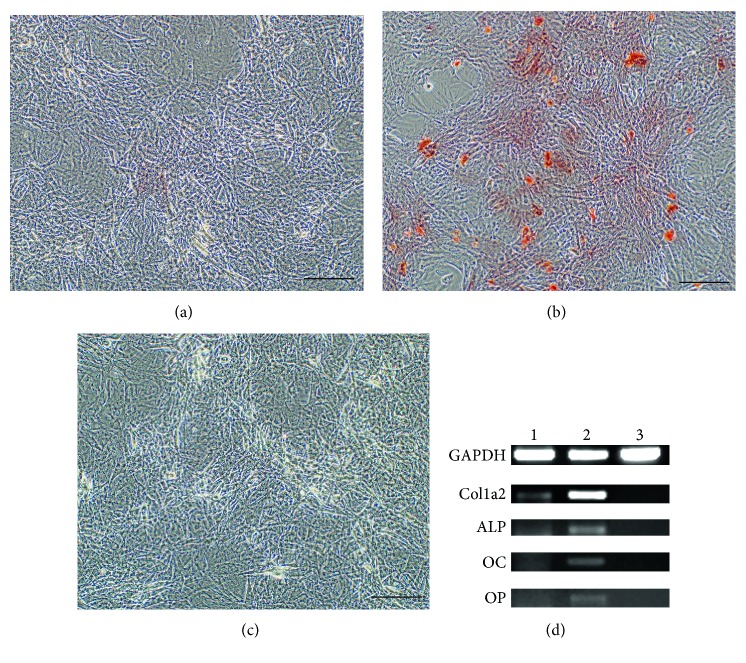
Analyses of the MSC sheets on the basis of Alizarin Red S staining and reverse transcription polymerase chain reaction (RT-PCR). Mineralization of MSC sheets was analyzed on the basis of Alizarin Red S staining in all groups. Osteogenic gene expression profiles in all groups of MSC sheets were analyzed on the basis of RT-PCR. MSC sheets maintained in osteogenesis-stimulating medium supplemented with the triple cocktail for 21 days stained positive with Alizarin Red S (b) and they exhibited increased levels of collagen type I (Col1a2), osteocalcin (OC), osteopontin (OP), and alkaline phosphatase (ALP) mRNA (d) in comparison to MSC sheets maintained in basal medium supplemented with only ascorbic acid (a, d). MSC sheets maintained in basal medium supplemented with only ascorbic acid showed slight osteogenic differentiation (a, d) when compared to the MSC sheets maintained in basal medium without any supplementation (c, d); however, this differentiation was not as strong as seen in the triple cocktail supplemented group (b, d). Sequences and sources of the primers used for the gene expression analyses were given in Table 1. (d) Lane 1: MSC sheets maintained in basal medium supplemented with only ascorbic acid; lane 2: MSC sheets maintained in osteogenesis-stimulating medium; lane 3: MSC sheets maintained in basal medium without any supplementation; GAPDH: glyceraldehyde 3-phosphate dehydrogenase (housekeeping gene used as loading control in this experiment). Scale bar = 200 *μ*m for (a, b, c).

It has been observed that MSC sheets cultured with basic medium supplemented with only 50 *μ*M L-ascorbic acid exhibited routinely a thicker and larger-surface cell sheet (Figures 1(a) and 1(c)) in comparison to those cultured with basic medium supplemented with all three components of the standard osteogenic cocktail described earlier (Figures 1(b) and 1(d)). MSC sheets maintained in osteogenesis-stimulating medium supplemented with the triple cocktail for 21 days stained positive with Alizarin Red S (Figure 2(b)), and they exhibited increased levels of collagen type I (Col1a2), osteocalcin (OC), osteopontin (OP), and alkaline phosphatase (ALP) mRNA (Figure 2(d)) in comparison to MSC sheets maintained in basal medium supplemented with only ascorbic acid (Figures 2(a) and 2(d)). These findings were in agreement with data from the literature [98]. MSC sheets maintained in basal medium supplemented with only ascorbic acid showed slight osteogenic differentiation (Figures 2(a) and 2(d)) when compared to the MSC sheets maintained in basal medium without any supplementation (Figures 2(c) and 2(d)); however, this differentiation was not as strong as seen in triple cocktail supplemented group (Figures 2(b) and 2(d)). Sequences and sources of the primers used for the gene expression analyses were given in Table 1. To be able to limit the unnecessary use of laboratory animals, MSC sheets maintained in basal medium supplemented with only ascorbic acid were considered as the control group for the rest of the experiments.

**Figure 3:**
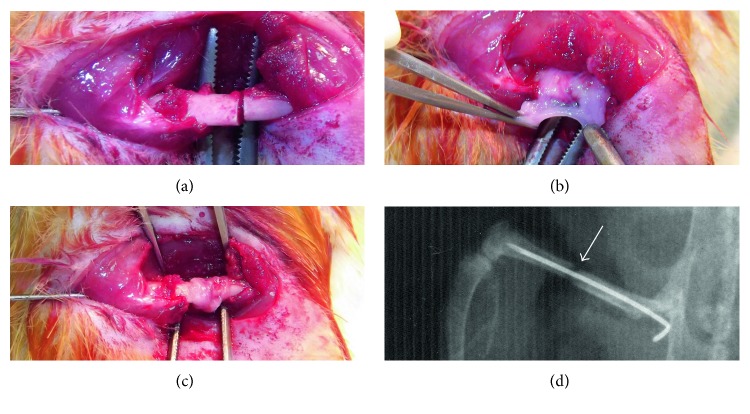
Application of MSC sheets onto the osteotomy sites in a rat fracture model. A 1 mm segmental bone defect was created by transverse osteotomy on rat femurs in this study (a). MSC sheets were wrapped around the fracture sites individually (b, c), and incisions on hind limbs were sutured. Postoperative control radiographs were taken after intramedullary fixation of fractures (d).

Since transplantable sheet size may matter in terms of repair of larger segmental bone defects, we tested the effectiveness of these two different types of MSC sheets in a rat femur fracture model described earlier [31]. As a modification to this model, to be able to follow the fracture healing process in time in all groups tested, we did not create a nonunion, instead, only created a 1 mm segmental bone defect by transverse osteotomy on the femurs in this study (Figure 3(a)). Loose internal fixation of the segmental bone defect was provided by a 21-gauge needle inserted into the intramedullary femoral shaft as described previously [31]. MSC sheets were wrapped around the fracture sites individually (Figures 3(b) and 3(c)), and incisions on hind limbs were sutured. The groups studied included (1) the osteotomy group without any sheets (sham group, *n* = 6), (2) the osteotomy group with MSC sheets induced with ascorbic acid only (control-sheet group, *n* = 6), and (3) the osteotomy group with MSC sheets induced with osteogenic cocktail (osteogenic-sheet group, *n* = 6). Unprotected weight bearing was allowed immediately after operation. Postoperative control radiographs were taken after intramedullary fixation of fractures (Figure 3(d)).

The biology of bone fracture healing has been described elsewhere as “It involves an acute inflammatory response including the production and release of several important molecules, and the recruitment of mesenchymal stem cells in order to generate a primary cartilaginous callus. This primary callus later undergoes revascularization and calcification, and is finally remodeled to fully restore a normal bone structure.” [103]. Thus, the histological stages to follow in our experiment can be summarized as inflammation followed by a primary cartilaginous callus formation (soft callus stage), bony callus formation (hard callus stage), and bone remodeling at the fracture site. During the follow-up of the operated rats, radiographs were taken every 2 weeks and 2 rats were sacrificed at each time point to examine the operated femurs in terms of gross and microscopic appearance.

**Figure 4:**
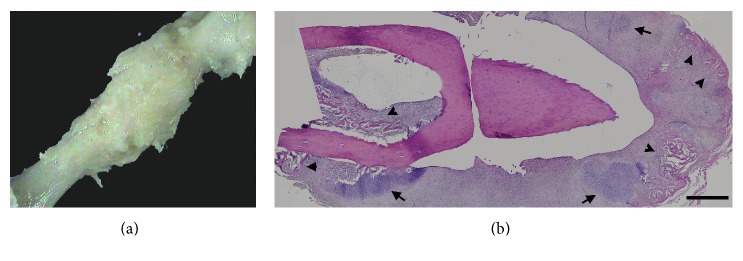
The gross (a) and microscopic (b) appearances of osteotomy site in the osteogenic-sheet group at the end of the second week. Callus formation was observed in the osteogenic-sheet group at the osteotomy site at the end of the second week (a). In microscopic examination, the osteogenic-sheet group exhibited a mainly cartilaginous (arrows) but also at some locations bony elements (arrow heads) containing callus formation at the end of the second week (b). Scale bar = 1 mm for (b).

**Figure 5:**
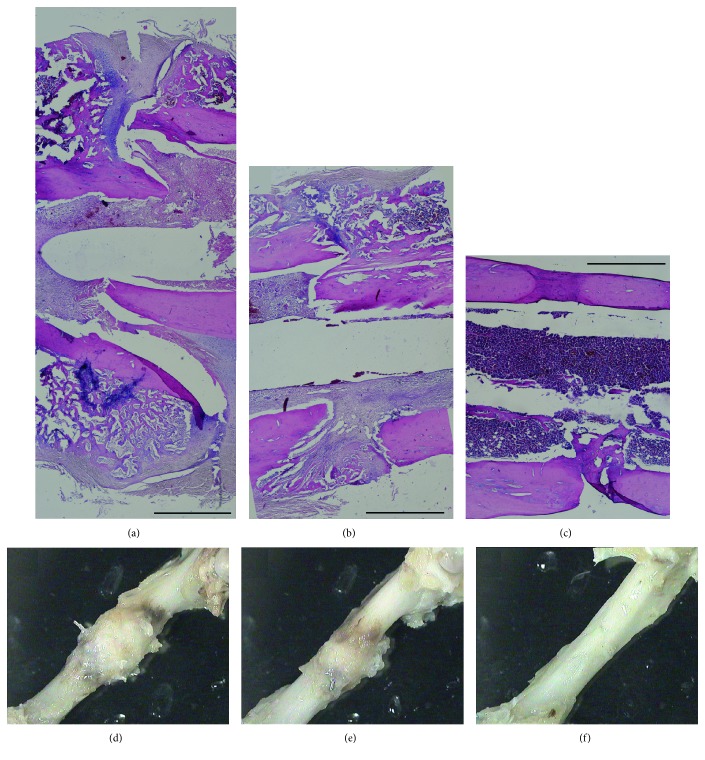
The gross (a, b, c) and microscopic (d, e, f) appearances of osteotomy sites in all three groups at the end of the fourth week. At the end of the fourth week, both sham (a and d) and control-sheet groups (b and e) exhibited bony callus at the fracture site with a slightly accelerated healing profile in the control group, that is, the callus size was smaller (e versus d) suggesting remodeling towards the healing process. However, in both sham and control-sheet groups, fracture ends were still apart from each other (a, b). In the osteogenic-sheet group (c, f), on the other hand, fracture site was already filled with bone tissue and fracture site entered to the bone remodeling stage. Scale bar = 1 mm for (a, b, c).

**Figure 6:**
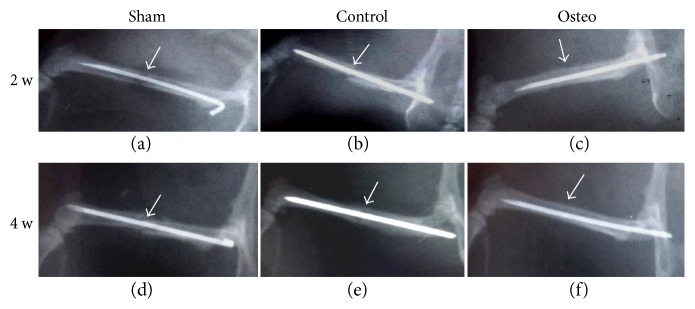
Radiographs taken at second (a, b, c) and fourth weeks (d, e, f) after MSC sheet transplantation. Radiographs supported the gross morphological and histological findings. Sham: operated but not treated with any MSC sheet. Control: operated and treated with MSC sheets maintained in basal medium supplemented with only ascorbic acid. Osteo: operated and treated with MSC sheets maintained in osteogenesis-stimulating medium.

Results showed that the osteogenic group exhibited a mainly cartilaginous but also at some locations bony elements encompassing callus formation at the end of the second week (Figures 4(a) and 4(b)). This finding suggested that the primary cartilaginous callus was formed even earlier in this group, and the examined callus at the end of the second week reached already to a transition stage from cartilaginous to bony callus. Callus formation in other two groups was observed at later stages. At the end of the fourth week, both sham (Figures 5(a) and 5(d)) and control groups (Figures 5(b) and 5(e)) exhibited bony callus at the fracture site with a slightly accelerated healing profile in the control group, that is, the callus size was smaller (Figures 5(e) versus 5(d)) suggesting remodeling towards the healing process. However, in both sham and control groups, fracture ends were still apart from each other (Figures 5(a) and 5(b)). In the osteogenic group, on the other hand, fracture site was already filled with bone tissue and the fracture site entered to the bone remodeling stage (Figures 5(c) and 5(f)). Radiographs taken at the second and fourth weeks supported these findings (Figure 6).

These preliminary findings indicate the importance of osteogenic induction process of these MSC sheets in vitro. Further studies are necessary to elucidate the best protocol for this induction process.

## 8. Conclusion

Taken this information together, tissue-derived MSC sheets can be used either in combination with various scaffolds/grafts or in scaffold/graft-free applications to shorten the treatment period in orthopaedics and traumatology cases involving nonunion, bone defects, osteonecrosis, and so forth. The in vitro osteogenic induction of these cell sheets is a necessary step accelerating the healing process described in bone tissue. The search for the best osteogenic induction combination and/or the application technique will hopefully continue for the years to come.

## Figures and Tables

**Table 1:** Primers for reverse transcription polymerase chain reaction (RT-PCR) analysis of gene expression.

Gene	GeneBank Acc. number	Primer sequence (5′-3′)	
GAPDH	NM_017008.3	F	GGGCTCTCTGCTCCTCCCTGT
		R	CATGGGGGCATCAGCGGAAGG
Collagen type I, alpha 2	NM_053356.1	F	AGCAGGTCCCCGAGGCAGAG
		R	GCAGGACCCGTTTGTCCGGG
Osteopontin	AB001382.1	F	TTGCCTGTTCGGCCTTGCCT
		R	ACGCTGGGCAACTGGGATGA
Alkaline phosphatase	NM_013059.1	F	CGGGTGAACCACGCCACTCC
		R	GGCCAGCAGTTCAGTGCGGT
Osteocalcin	M25490.1	F	TCCTGGGGTTTGGCTCCTGCT
		R	GGCGAAGGCCTGGAAGGGGA

F: forward primer; R: reverse primer.
